# Tissue stiffness heterogeneity in the jaw and temporomandibular joint: its impact on tumor metabolism and considerations for *in vitro* model development

**DOI:** 10.3389/fphys.2025.1661054

**Published:** 2025-10-09

**Authors:** Lingjie Li, Ping Deng, Siyu Hou, Guangyue Li, Min Suo, Ling Xu, Chao Wang, Jinlin Song

**Affiliations:** ^1^ College of Stomatology, Chongqing Medical University, Chongqing, China; ^2^ Department of Prosthodontics, Stomatological Hospital of Chongqing Medical University, Chongqing, China; ^3^ Chongqing Municipal Key Laboratory for Oral Biomedical Engineering of Higher Education, Chongqing, China; ^4^ College of Stomatology, Xi’an Jiaotong University, Xian, China; ^5^ Beijing Advanced Innovation Center for Biomedical Engineering, Beihang University, Beijing, China

**Keywords:** stiffness, heterogeneity, metabolism, bone- and cartilage-forming tumors, *in vitro* model

## Abstract

Malignant bone- and cartilage-forming tumors exhibit heterogeneous clinical behavior across various body regions. Understanding the mechanisms underlying these differences is essential for developing targeted diagnostic and therapeutic strategies. This review proposes the hypothesis that tissue stiffness heterogeneity contributes to the distinct progression and prognosis of tumors in the jaw and temporomandibular joint (TMJ) compared to peripheral skeletal sites, potentially through stiffness-mediated metabolic reprogramming. To evaluate this hypothesis, a conceptual framework is provided to guide future research. This review summarizes spatial and temporal variations in stiffness across the jaw, TMJ, and femur, and introduces potential mechanisms through which mechanical stiffness may influence tumor metabolism. Technical strategies and material considerations for designing scaffolds that mimic bone and cartilage stiffness are discussed, along with current applications of stiffness-biomimetic scaffolds for *in vitro* investigation of malignant bone- and cartilage-forming tumors. By integrating insights from mechanobiology, tumor metabolism, and scaffold engineering, this review aims to facilitate the development of targeted experimental approaches that may contribute to more effective treatment strategies.

## Introduction

According to statistics, the maxillofacial region is the most common site for malignant bone- and cartilage-forming tumors in the head and neck (Pellitteri et al., 2007; Stewart et al., 2014). The complex anatomy and microenvironment of this region, particularly the jaw and TMJ, pose significant challenges for the diagnose and treatment. The jaw is the fourth most common site for osteosarcoma (OS). OS in the jaw is predominantly of the chondroblastic variant, whereas the osteoblastic variant is more common in long bones. OS in the jaw has a lower tendency to metastasize compared to extragnathic sites, with local progression being the primary cause of morbidity and mortality (Stewart et al., 2014; Seng et al., 2019). Chondrosarcoma (CS) in the maxillofacial region accounts for less than 3% of all cases. The incidence rate is higher in the maxilla compared to the mandible, with the high incidence site corresponding to the region of endochondral ossification (Ram et al., 2013). CS of the TMJ is rare compared to other sites, but it exhibits a lower recurrence rate and higher survival rate, albeit with an earlier onset age compared to general CS (Faro et al., 2021). Mesenchymal CS, a variant of CS known for its high invasiveness, commonly affects the jaw, making it the most frequent site in the head and neck region (Stanbouly et al., 2021). Compared to CS found in long bones, mesenchymal CS that occurs in the head and neck bones demonstrates faster growth, recurrence, and metastasis rates (Flaman et al., 2020; Choo et al., 2019). Currently, treatment strategies for malignant bone- and cartilage-forming tumors in the maxillofacial region are generally similar to those used in other body regions. However, the prognosis and long-term survival data are not entirely consistent with other sites. For instance, the use of neoadjuvant chemotherapy in improving the prognosis of OS in the jaw remains controversial compared to its application in long bones (Seng et al., 2019). Despite numerous studies observing and summarizing the development patterns of tumors at different anatomical positions, the specific reasons for these differences remain unclear.

Previous studies have shown that mechanical signals from surrounding tissues can influence tumor growth and distant migration (Nia et al., 2020; Deng et al., 2022). Accordingly, the material properties of tissues may contribute to differences in tumor progression and prognosis. Stiffness is an important parameter for evaluating tissue material properties (Jiang et al., 2022). Measurements indicate that the stiffness of temporomandibular condylar cartilage and articular disc is less than one percent of that of the jaw bone (Lettry et al., 2003; Wright et al., 2016). Furthermore, spatial and temporal heterogeneity in stiffness exists across different anatomical sites, even within the same type of tissue (Wright et al., 2016). Variations in stiffness have also been documented among different regions of the same bone (Bayraktar et al., 2004). Additionally, bone stiffness undergoes dynamic changes with maturation and aging (Hart et al., 2020; Lu et al., 2012), which may further influence tumor behavior and clinical outcomes. Although direct evidence linking stiffness to the distinct development and prognostic features of malignant bone- and cartilage-forming tumors in the jaw and TMJ remains limited, a growing body of studies highlights the regulatory effect of stiffness in these tumors. Current research primarily focuses on OS. In three-dimensional culture systems, OS cells exhibit sensitivity to scaffold stiffness. Evidence shows that increased stiffness promotes the expression of tumor angiogenesis-related factors (hypoxia-inducible factor 1-alpha(HIF-1α) and vascular endothelial growth factor (VEGF)), tumorigenesis-related matrix metalloproteinases (MMPs), as well as metastasis and invasion-related markers such as ALDH and CD133 (Lin et al., 2023; Jiang et al., 2019). These effects are mediated through adhesion and mechanotransduction factors, including paxillin, FAK (Jiang et al., 2019), integrin α5, and MAPK (Lin et al., 2023). Variations in scaffold stiffness can also modulate therapeutic resistance in OS (Chim et al., 2023; Molina et al., 2019). Higher stiffness may enhance resistance to combination therapy targeting IGF-1R/mTOR, potentially through mechanosensitive pathways involving YAP/TAZ (Molina et al., 2019). Nevertheless, direct evidence correlating jaw and TMJ stiffness with malignant bone- and cartilage-forming tumors is still lacking. Elucidating the influence and underlying mechanisms of jaw and TMJ stiffness on tumor progression may provide a theoretical foundation for improving clinical diagnosis and treatment.

In addition, one such potential mechanism linking tissue stiffness to tumor progress and prognosis is metabolic reprogramming, a process that could be driven by biomechanical cues from the extracellular matrix (ECM). This reprogramming includes, but is not limited to, the Warburg effect, alterations in mitochondrial activity, and the changes in the synthesis and metabolism of amino acids and lipids (Jiang et al., 2022; Vander Heiden and DeBerardinis, 2017). These processes have also been observed in malignant bone- and cartilage-forming tumors and are being investigated as potential therapeutic targets (Miallot et al., 2021; Jiménez et al., 2022; Micaily et al., 2021). Therefore, differences in tumor development and treatment outcomes between the jaw and TMJ compared to other sites may arise from stiffness-mediated variations in tumor metabolism. However, evidence supporting this relationship requires further investigation.

It is worth noting that research on the relationship between tissue stiffness heterogeneity and tumor metabolism faces two major challenges. Firstly, the stiffness range of most *in vitro* models constructed in research is between kPa and MPa, which is far lower than the stiffness range of cartilage and bone (MPa-GPa). Secondly, although some scholars propose that material stiffness at the GPa level regulates tumor progression, the impact of stiffness on tumor cells seems to lose significance at varying GPa levels (Ruppender et al., 2010). Given the limited differences in stiffness within the same tissue type from different regions, it raises the question of whether the characteristics of malignant bone- and cartilage-forming tumors in the jaw and TMJ are truly related to the surrounding tissue stiffness. Interestingly, a new study suggests that cells can perceive differences in material stiffness within the GPa range. This report showed that immortalized mesenchymal stem cells display significantly greater spreading areas when cultured on poly ((tetrahydropyran-2-yl N-(2 methacryloxyethyl) carbamate)-b-(methyl 4-(3-methacryloyloxypropoxy) cinnamate)) films with 28 GPa compared to 19 GPa. In addition, the higher the stiffness, the flatter the cells, and the attachment is enhanced (Zanut et al., 2023). Given that the cytoskeleton is closely associated with tumor metabolism (Park et al., 2020; Wang EJ. et al., 2022), it follows that although differences in bone or cartilage stiffness across different regions may be minimal, they could still have distinct impacts on tumor metabolism. Therefore, a deeper understanding of the effects of time and space on bone and cartilage stiffness, and using these data to develop more tissue stiffness biomimetic scaffolds for tumor research, is highly valuable.

This review proposes that stiffness heterogeneity may contribute to the distinct progression and prognosis of malignant bone- and cartilage-forming tumors in the jaw and TMJ, potentially through its regulation of tumor metabolism, though conclusive evidence remains to be established. To help elucidate these potential relationships, we emphasize the need to characterize the stiffness properties of the jaw and TMJ as a basis for designing biomimetic scaffolds suitable for *in vitro* study. The article introduces the spatial and temporal heterogeneity in these regions and discusses how stiffness variations may influence tumor metabolic reprogramming. Furthermore, it discusses the key considerations involved in constructing stiffness biomimetic scaffolds for studying tumor metabolism and summarizes recent progress in applying such models to investigate malignant bone- and cartilage-based tumors.

## Heterogeneity of jaw stiffness

### Spatial heterogeneity of jaw stiffness

Approximately 90% of the bone ECM is composed of collagen, primarily type I collagen. Additionally, other proteins such as osteocalcin, osteopontin, and proteoglycans play crucial roles in facilitating the deposition of hydroxyapatite (HA) and the mineralization of collagen. These processes enhance the tensile modulus of bone, improve the energy dissipation, and increase the resistance to fractures (Nair et al., 2013; Zhu et al., 2021). The mechanical properties of bone exhibit significant spatial heterogeneity due to variations in composition and structure, which regulated with factors such as species, age, disease (Rezaei et al., 2019; Morgan et al., 2003; Romme et al., 2013). Cortical bone accounts for approximately 80% of the bone tissue, while cancellous bone makes up the remaining 20% (Hart et al., 2020). Cancellous bone is characterized by its porous structure, which shows lower calcium content, and an elastic modulus that is approximately 10% lower than that of cortical bone (Bayraktar et al., 2004). It should be noted that in different reports, the modulus may differ significantly (Table 1), up to three times, depending on the measurement site and method used (Morgan et al., 2003; Romme et al., 2013; Wu et al., 2018).

**TABLE 1:** Spatial heterogeneity of bone stiffness (Distribution locations).

Measurement method	Measurement position	Femur	Maxilla	Mandible	Mandibular condyle
Tension	Cortical	19.9 ± 1.8 GPa (Bayraktar et al., 2004)	—	4.3 GPa–10.1 GPa (Lettry et al., 2003)	—
	Trabecular	18.0 ± 2.8 GPa (Bayraktar et al., 2004)	—	—	—
Nanoindentation	Cortical	(15.8 ± 5.3) GPa- (21.2 ± 5.3) GPa (Zysset et al., 1999)	14.5 GPa–15.3 GPa (Cortical + Trabecular) (Seong et al., 2009)	16.8 GPa–19.7 GPa (Cortical + Trabecular) (Seong et al., 2009)	7.48 ± 3.09 GPa (Kim et al., 2015)
	Trabecular	11.4 ± 5.6 GPa (Zysset et al., 1999)	15.4 GPa (Average of Mandible and Maxilla) (Seong et al., 2009)		5.11 ± 2.82 GPa (Kim et al., 2015)
Compression	Cortical	15–20 GPa (Öhman et al., 2011)	—	96.2 ± 40.6 MPa (Cortical + Trabecular) (Misch et al., 1999)	—
	Trabecular	(2.54 ± 0.22) GPa-(3.47 ± 0.41) GPa (Hong et al., 2007)	—	56.0 ± 29.6 MPa (Misch et al., 1999)	(90 ± 101) MPa-(685 ± 338) MPa (van Eijden et al., 2006)
Ultrasonic method	Cortical	(25.23 ± 3.57) GPa- (32.51 ± 0.87) GPa (Hunt et al., 1998)	(6.9 ± 1.1) GPa-(18.7 ± 3.4) GPa (Peterson et al., 2006)	(12.7 ± 1.8) GPa-(22.8 ± 5.4) GPa (Schwartz-Dabney et al., 2003)	—
	Trabecular	13.0 ± 1.47 GPa (Ashman and Rho, 1988)	—	—	—

The jaw, comprising the maxilla and mandible, is a critical component of the maxillofacial structure. Studies have demonstrated that its matrix composition ratio differs from extramaxillofacial bones, such as limb bones. For instance, the mandible contains a higher percentage of collagen compared to the long bone (Matsuura et al., 2014), and the collagen cross-link profiles and mineralization vary as well (Romanowicz et al., 2022). The jaw exhibits fewer mature cross-links and hydroxylation of lysine, leading to more frequent degradation and renewal (Matsuura et al., 2014). Consequently, the mechanical properties of bone at different distribution locations may vary. Table 1 presents some measurement data regarding the moduli of the human femur, maxilla, mandible, and mandibular condyle. The data indicate that, compared to the femur, the jaw exhibits lower tensile, elastic, and compression moduli. Furthermore, the moduli of the cortical bone are higher than those of the trabecular bone, regardless of whether it is in the femur or the jaw. In the jaw, material properties also differ among various bone blocks in the maxillofacial region. The maxilla has lower elastic and shear moduli than the mandible (Gharpure et al., 2016). In contrast to the mandible, the maxilla exhibits lower porosity and bone turnover rates (Huja et al., 2006), which correspond to a lower elastic modulus (Seong et al., 2009).

The mechanical properties of the jaw also display heterogeneity based on anatomic locations (Table 2). In the maxilla, the elastic modulus of the cortical bone in the palate is higher than that of the alveolar ridge (Schwartz-Dabney and Dechow, 2002), while the area with the highest measurement value for elastic modulus is located at the junction of the maxilla and zygoma (Peterson et al., 2006). In the mandible, the elastic and shear moduli of the cortical bone in the alveolar bone are lower than those of the basal bone, and there is no significant difference between the buccal and lingual sides (Zapata et al., 2011). Furthermore, although a direct comparative study is lacking, data obtained from different literature sources suggest that the elastic modulus of the condyle may be smaller than that of the mandibular body (Seong et al., 2009; Kim et al., 2015).

**TABLE 2:** Spatial heterogeneity of bone stiffness (Anatomic locations).

Anatomic locations	Measurement method	Measurement position	Compare	References
Femur	Element/Finite element model	Cortical	Femoral head > Intertrochanteric region	Morgan et al. (2003), Yoon et al. (2021)
Maxilla	Nano-indentation	Cortical + Trabecular	Posterior region > Anterior region	Seong et al. (2009)
	Ultrasonic method	Cortical	Zygomaticomaxillary suture > Palate > Alveolar	Peterson et al. (2006), Dechow et al. (2010)
Mandible	Nano-indentation	Cortical + Trabecular	Posterior region > Anterior region	Seong et al. (2009)
	Ultrasonic method	Cortical	Ramus > Corpus; Facial corpus > Lingual corpus	Schwartz-Dabney et al. (2003)
	Compression	Trabecular	Anterior region > Middle and distal regions	Misch et al. (1999)
Mandibular condyle	Discrete element/Finite element (DE/FE) model	Trabecular	Superolateral region > Superomedial and Inferolateral region > Inferomedial region	van Eijden et al. (2006)

### Temporal heterogeneity of jaw stiffness

The age-related changes in the jaw encompass multiple aspects. Table 3 provides some measurement data regarding the temporal heterogeneity of human bone stiffness. During the maturation of cortical bone, there is an increase in thickness, density, and stiffness, while anisotropy decreases (Wang et al., 2010). Although aging leads to a decrease in cortical density and an increase in jaw porosity, it may not necessarily be associated with tooth loss (Atkinson and Woodhead, 1968). There are differing viewpoints on how tooth extraction can affect the thickness, density, modulus, and directional orientation of the craniofacial cortical bone, with some studies suggesting that the degree of trabecular resorption based on tooth loss is greater compared to cortical bone (Dechow et al., 2010; Bertl et al., 2015). Additionally, age-related changes in bone are not entirely consistent across different positions. The mandible exhibits a faster growth rate than the maxilla during adolescence (Nahhas et al., 2014). The volume of alveolar bone between teeth roots may increase with age, while the tibia demonstrates an age-dependent decrease in trabecular amount and subchondral bone mass (Nenda et al., 2016). Interestingly, a report suggests that aging, as a single factor, may not have a significant impact on the material properties of the mandibular condyle (Kim et al., 2015). From these discussions, it becomes evident that the temporal heterogeneity of jaw material properties is complex and controversial, necessitating further research in the future.

**TABLE 3:** Temporal heterogeneity of bone stiffness (Anatomic locations).

Anatomic locations	Measurement method	Measurement position	Compare	References
Femur	Microindentation/Compression	Cortical	Adult > Child	Öhman et al. (2011), Semaan et al. (2019)
	Nanoindentation/Ultrasonic method	Cortical	Aged > Adult	Singleton et al. (2021), Malo et al. (2013)
	Tension	Trabecular	No significant relationship with osteoporosis and age	Peters et al. (2018), Frank et al. (2021)
Maxilla	Nanoindentation	Cortical	Dentate individuals > Edentate individuals	Dechow et al. (2010)
Mandible	Ultrasonic method	Cortical	Lingual corpus, facial corpus, and lingual condylar neck: Edentate > Dentate; Ramus: Dentate > Edentate	Schwartz-Dabney and Dechow (2002)
Mandibular condyle	Nanoindentation	Cortical + Trabecular	No significant relationship with age	Kim et al. (2015)

The temporal heterogeneity of bone stiffness is primarily influenced by hormones and mechanical forces. Hormones play a crucial role in regulating the growth, development, and stability of bones. Age-related changes in growth, gonadal, and calciotropic hormones result in fluctuations in bone metabolism, which dynamically regulate bone formation and absorption, ultimately contributing to age-related bone diseases such as osteoporosis (Hart et al., 2020). However, the impact of hormones on bone homeostasis varies depending on the distribution location. Compared to long bones, the jaw is less responsive to changes in estrogen levels (Mavropoulos et al., 2007). Some studies have suggested that abnormal expression of 1,25-dihydroxyvitamin D3 and parathyroid hormone can affect bone mineralization and osteoclast production in long bones, but the influence of parathyroid hormone on the jaw bone remains controversial (Liu et al., 2009; Chaichanasakul et al., 2014; Chen et al., 2017).

On the other hand, tooth loss can lead to changes in oral function, which in turn can alter the morphology and material properties of the jaw. Studies have shown that jaw remodeling is more active compared to long bones, possibly due to the higher forces exerted on the jaw during chewing compared to the forces experienced by the leg during walking (Nenda et al., 2016). Furthermore, different types of forces act on the maxilla and mandible during chewing. The maxilla primarily withstands compressive forces that resist chewing, while the mandible is subjected to bending and twisting forces (Huja et al., 2006; Daegling and Hylander, 1997). Consequently, after tooth loss, the loading mode changes, resulting in asynchronous changes in the volume, density, and mechanical and physical properties of the craniomaxillofacial skeleton. Compared to a dentate jaw, individuals with an edentulous jaw show increased cortical bone stiffness above the orbit, but reduced maxilla stiffness (Dechow et al., 2010). Additionally, the rate of alveolar bone absorption after tooth loss is four times higher in the mandible compared to the maxilla. Despite this, the elastic modulus and hardness of the mandible after tooth loss remain higher than those of the maxilla. Meanwhile, the anterior segment (original anterior tooth area) of both the maxilla and mandible is smaller than the posterior segment (original posterior tooth area) (Seong et al., 2009).

## Heterogeneity of TMJ cartilage stiffness

### Spatial heterogeneity of TMJ cartilage stiffness

Malignant tumors involving the TMJ, such as CS, are extremely rare (with the mandibular condyle being the primary origin). However, tumor symptoms and signs often overlap with TMJ dysfunction, leading to diagnostic challenges. Moreover, surgical operation can greatly affect the patient’s appearance and ability to consume food (Yibulayin et al., 2020). Therefore, precise diagnosis and early treatment are necessary.

The TMJ is not completely consistent with other joints in terms of its structure. Not only is it covered with cartilage on the surface of the mandibular condyle and articular tubercle of the temporal bone, but it also features a disc with uneven thickness that separates the articular surfaces (Kuroda et al., 2009). Table 4 presents comparative data on the cartilage moduli of the distal femoral condyle, mandibular condyle, and TMJ disc, including human and porcine cartilage (the mechanical properties of porcine and human cartilage are similar). For instance, the tensile modulus of the mandibular condyle cartilage and disc is higher than that of the distal femoral condylar cartilage (Wright et al., 2016; Silver et al., 2002; Singh and Detamore, 2008), whereas the compressive modulus exhibits an opposite trend (Kabir et al., 2021; Juran et al., 2013; Kalpakci et al., 2011).

**TABLE 4:** Spatial heterogeneity of cartilage stiffness (Distribution locations).

Measurement method	Distal femoral condylar cartilage	Mandibular condylar cartilage	Temporomandibular joint disc
Tension	1.492–6.29 MPa (Silver et al., 2002)	(10.1 ± 5.5) MPa-(24 ± 12) MPa (Singh and Detamore, 2008)	(11.2 ± 6.8) MPa-(14.3 ± 8.5) MPa (Wright et al., 2016)
Compression	10.60 ± 3.62 MPa (Kabir et al., 2021)	(0.3850 ± 0.001) MPa-(1.1950 ± 0.036) MPa (Porcine) (Juran et al., 2013)	(1.116 ± 0.153) MPa-(4.800 ± 3.597) MPa (Kalpakci et al., 2011)
Shear	0.77 ± 0.62 MPa (Peters et al., 2018)	(0.115 ± 0.0832) MPa-(12.400 ± 6.193) MPa (Porcine) (Gologorsky et al., 2021)	0.97 MPa–2.7 MPa (Lai et al., 1998)

Compositional differences and changes in the ECM are significant factors contributing to the spatial stiffness heterogeneity of cartilage. Studies have revealed that the adult articular cartilage microenvironment contains a small proportion of chondrocytes compared to a larger proportion of ECM. The solid phase components of the ECM primarily consist of abundant collagen (mainly type II) and proteoglycans (Lu et al., 2009). Type II collagen serves as the primary protein that maintains the integrity of the cartilage ECM, while the interaction between proteoglycans and collagen provides excellent compressive strength (Krishnan and Grodzinsky, 2018; Peng et al., 2021). Moreover, the involvement of other collagen families in the mechanics of cartilage ECM has also been reported. For example, the dissipation of type III and V collagens leads to an increase in cartilage thickness and a decrease in tissue modulus (Wang et al., 2020; Chandrasekaran et al., 2021).

Another structure involved in transmitting biomechanical signals is the pericellular matrix (PCM). Studies have shown that the composition and mechanical properties of the PCM of articular cartilage are not entirely consistent with those of the ECM. The PCM has a thickness of approximately 2–4 μm surrounding chondrocytes, which may act as a buffer zone for chondrocytes to receive external mechanical stimuli. The PCM is rich in type VI collagen, fibronectin 1, and perlecan, among other components (Wilusz et al., 2014; Guilak et al., 2018). The elastic modulus of the PCM is 27–205 kPa, which is lower than the order of magnitude of the ECM (MPa) (Wilusz et al., 2014). It is important to note that alterations in the PCM can affect the overall mechanical properties of cartilage. For instance, data has suggested that the quantity of perlecan and type VI collagen in the PCM may contribute to the uneven distribution of cartilage elastic modulus and degenerative changes in the articular surface (Wilusz et al., 2012; Xu et al., 2016).

In addition to variations in cartilage moduli across different distribution locations, there is also an uneven distribution of moduli within the cartilage itself. As depicted in Table 5, differences in moduli can be observed on the superficial and deep regions of the cartilage, as well as in the central and surrounding areas. This heterogeneity might be also influenced by microenvironment components. Histologically, the cartilage of mandibular condylar cartilage can be divided into four zones, from the articular surface to the bone surface: the fibrous, proliferative, mature and hypertrophic zones (Kuroda et al., 2009; Mizoguchi et al., 1996). Each zone has a unique composition ratio of ECM and exhibits varying degrees of mechanical property heterogeneity. The collagen in the fibrous zone is mainly type I, tightly arranged and parallel to the articular surface. Meanwhile, the fibrous zone lacks proteoglycans. The proliferative zone serves as a cell bank and is rich in collagen. The collagen in the mature and hypertrophic zones is mainly type II, and the amount of proteoglycan in the hypertrophic zone is the highest among the four zones (Kuroda et al., 2009; Korhonen et al., 2002; Gologorsky et al., 2021). Correspondingly, the difference in shear modulus between each zone can be up to a hundred times, and the shear modulus of the fibrous and mature zones is relatively lower than the other two zones (Gologorsky et al., 2021). In addition, the mechanical properties of other cartilage in the TMJ also show the heterogeneity of stiffness. For instance, the collagen in the disc is mainly type I, and the elastic moduli of the anterior, intermediate, and posterior zones are inconsistent, with the intermediate zone having the highest elastic modulus (Tanne et al., 1991; Kim et al., 2003).

**TABLE 5:** Spatial heterogeneity of cartilage stiffness (Anatomic locations).

Anatomic locations	Measurement method	Compare	References
Distal femoral condylar cartilage	Compression/Nanoindentation/Shear	deepest region > Superficial region	Chen et al. (2001), Antons et al. (2018), Henak et al. (2016)
	Tension	Superficial region > deepest region	Kempson (1991)
	Nanoindentation	Lateral region > Medial region	Peters et al. (2018)
Mandibular condylar cartilage	Shear	deepest region (Porcine) > Superficial region	Gologorsky et al. (2021)
	Tension	Central region > other region > Anterior region	Singh and Detamore (2008)
	Compression/Shear	Central region > Anterior and posterior region (Porcine)	Juran et al. (2013)
Temporomandibular joint disc	Tension	Anteroposteriorly: Central region > Medial and Lateral regions; Mediolaterally: Posterior region > Central and Anterior region; Central region: Anteroposteriorly > Mediolaterally	Wright et al. (2016)
	Compression/Tension	Anterior band > Posterior band	Gutman et al. (2018)

### Temporal heterogeneity of TMJ cartilage stiffness

Similar to bone, the moduli of cartilage also undergo changes as age (Table 6). Chondrocytes, which are responsible for producing the ECM of cartilage, play a crucial role in the formation and degradation of cartilage. As cartilage mature and age, there are alterations in chondrocyte number, phenotype, and mechanical sensitivity. These changes subsequently affect various aspects of the ECM, including its quantity, morphology, size, structure, and other components (Martin and Buckwalter, 2001; Chen PJ. et al., 2020; Fan et al., 2022). With advancing age, there is a shift from cartilage to bone replacement in the TMJ cartilage. This is characterized by an increase in the volume fraction and density of the condylar bone, while the thickness of the cartilage decreases. In younger individuals, the mandibular condylar cartilage and subchondral bone exhibit active differentiation of osteoclasts. However, in aging individuals, there are fewer osteoclasts and cartilage collagen cells in the subchondral bone. Additionally, there is a loss of aggrecan from ECM and an increase in collagen fibers migrating towards the cartilage surface. These changes are accompanied by the migration of the mineralization front towards the uncalcified layer of the cartilage (Chandrasekaran et al., 2021; Chen PJ. et al., 2020). In the end, the age-dependent remodeling of the ECM described above leads to a decrease in the shear modulus of cartilage and an increase in the elastic modulus of the subchondral bone (Peters et al., 2018).

**TABLE 6:** Temporal heterogeneity of cartilage stiffness (Anatomic locations).

Anatomic locations	Measurement method	Compare	References
Distal femoral condylar cartilage	Tension	Aged > Adult and child	Kempson (1991)
	Nanoindentation/Finite element/Tension	Normal person > Patients with osteoarthritis	Silver et al. (2002), Ebrahimi et al. (2021)
Mandibular condylar cartilage	Compression	Artificial aging by ribose > Normal (Porcine)	Mirahmadi et al. (2018a)
Temporomandibular joint disc	Shear	Aged > Adult	Lai et al. (1998)

The aging-related changes in the material properties of TMJ cartilage are regulated by mechanical signals. Mechanical-sensitive proteins, including Indian Hedgehog, the Wnt/β-catenin pathway, and other signaling pathways, mediate biological signal transduction during chondrogenesis. When the mechanical force is lost, the generation of new cartilage is inhibited. Aging leads to alterations in occlusion, muscle function, and joint friction, which may contribute to the process of cartilage aging through changes in mechanical forces (Chen PJ. et al., 2020; Sobue et al., 2011; Nickel et al., 2018). It is worth noting that the impact of mechanical force on cartilage remodeling also depends on its spatial distribution. For example, when the surface layer of cartilage bears a load, it becomes more susceptible to mechanical damage, resulting in increased protein hydrolysis activity and significant changes in mechanical and physical properties (Lotz and Loeser, 2012).

In addition to the mechanical force, hormone can also influence the material properties of cartilage. Studies have suggested that supplementing parathyroid hormone-related proteins can alleviate the accumulation of aging cells in the condyle and promote the proliferation of bone marrow mesenchymal stem cells (Cui et al., 2020). Excessive growth hormone delays the maturation of condylar chondrocytes and increases endochondral ossification (Ramirez-Yañez et al., 2004). Moreover, maintaining the homeostasis of estrogen is essential for ensuring the integrity of TMJ. Excessive estrogen leads to premature stagnation or even degeneration of TMJ development in young rats, while deficiency in estrogen leads to TMJ degeneration in adult rats (Chen J. et al., 2014; Wang et al., 2013; Yadav et al., 2018).

During the generation and remodeling of cartilage, the compressive and tensile moduli initially increase and then decrease with age, reaching their highest levels during juvenile stages. The stiffness of juvenile cartilage is 4.7 times greater than that of newly formed cartilage and 3.5 times greater than that of mature cartilage (Bielajew et al., 2022). However, the changes in cartilage material properties associated with aging remain controversial. Research has shown that, although not always exhibiting clinical symptoms, the degeneration level of TMJ increases between the ages of 60 and 70 (Yadav et al., 2018). Aging leads to an increase in the compressive stiffness of condylar cartilage due to higher levels of collagen crosslinking (Mirahmadi et al., 2018a). Meanwhile, there is also data suggesting that aging may not necessarily be reflected in the stiffness characteristics of TMJ condylar cartilage (Armstrong and Mow, 1982; Mirahmadi et al., 2018b). This contradiction may be attributed to differences in research species and measurement methods.

It is worth noting that a critical consideration when interpreting and comparing reported stiffness values of jaw, TMJ, and other tissues is the substantial methodological heterogeneity across studies. The mechanical properties of biological tissues are highly sensitive to measurement techniques, which vary widely in their principles, spatial resolution, and testing conditions. For instance, data obtained from nanoindentation can vary significantly depending on sample hydration state, probe geometry, and the analytical model used for data processing (Rodriguez-Florez et al., 2013; Armitage and Oyen, 2017). Similarly, techniques such as ultrasonic methods and mechanical testing of bulk specimens each operate under distinct contact mechanisms, strain rates, and environmental controls (Currey, 2009; Gallant et al., 2013). All these factors collectively influence the resulting modulus values.

This methodological diversity poses a significant challenge for drawing direct comparisons of absolute stiffness values between craniofacial and peripheral skeletal and cartilaginous sites based on literature data. Variations may arise not only from anatomical and biological factors but also from technical discrepancies. Furthermore, sample preparation methods, such as dehydration, embedding, or testing under hydrated conditions, can further alter measured mechanical outcomes. Therefore, while the collected data suggest trends in stiffness variations across sites, the lack of a standardized measurement framework necessitates cautious interpretation of quantitative comparisons. Future studies should employ consistent, multimodal approaches across anatomical regions will be essential to establish more definitive mechanistic links between tissue level mechanics and tumor development.

## The potential association between tissue stiffness and bone- and cartilage-forming tumor metabolism

**FIGURE 1:**
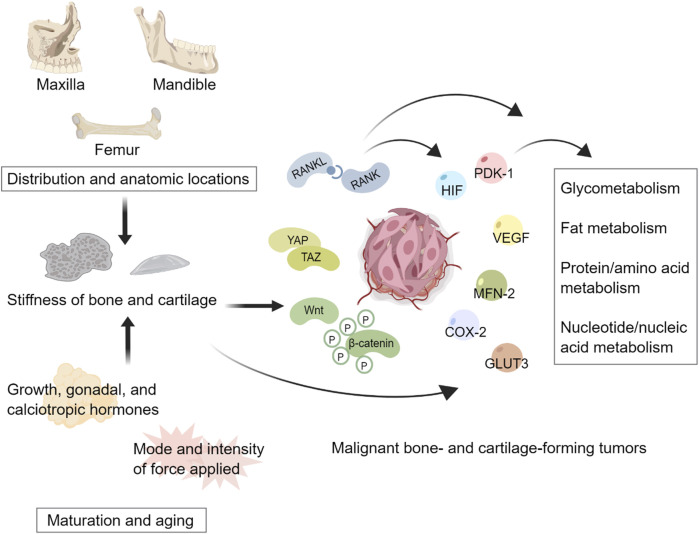
Tissue stiffness heterogeneity regulates metabolism of malignant bone- and cartilage-forming tumors. Distributions and anatomic locations contribute to the spatial heterogeneity of bone and cartilage stiffness. Changes in hormones and forces acting on tissues result in the temporal heterogeneity of bone and cartilage stiffness. Stiffness heterogeneity can potentially mediate multiple mechanosensitive signaling pathways to regulate the metabolism of tumors. This figure created with MedPeer.cn.

As illustrated in Figure 1, the stiffness of bone and cartilage can modulate the metabolism of bone- and cartilage-forming tumors through multiple mechanosensitive signaling pathways.

### Spatial heterogeneity of bone stiffnesses and tumor metabolism

The proliferation, osteogenic differentiation, and mineralization of stem cells and osteoblasts exhibit variation across different anatomical sites (Stefanik et al., 2008; Aghaloo et al., 2010; Eber et al., 2021). For instance, osteoblasts derived from the jaw demonstrate a higher rate of proliferation and express higher levels of VEGF compared to those derived from long bones in mice. This distinction contributes to the increased capacity of jaw-derived osteoblasts to promote tumor cell growth (Eber et al., 2021). These cellular processes involve intricate metabolic changes, which may also differ among anatomical sites (Hua et al., 2018; Lekvijittada et al., 2021). A recent study has provided evidence for the spatial heterogeneity of metabolism in the femur. Anabolic pathways, including mannose type‐O glycan biosynthesis and linoleic metabolism, are regulated by the varying bone elastic modulus at different positions along the femur (Welhaven et al., 2022). However, it remains uncertain whether a similar phenomenon exists in the jaw.

A recent report by Olivier Cuvillier et al. suggested that the metastasis rate of OS from jaw is lower than that of OS occurring in long bones, accompanied by reduced levels of hypoxia markers glucose transporter protein (GLUT)-1, SphK1, and S1P1 (Gomez-Brouchet et al., 2022). However, it remains unclear whether these findings are directly linked to the characteristics of tissue materials. Due to the lack of comparative data, relying solely on information from other bone sites may not provide a comprehensive understanding of the specific characteristics of tumors occurring in the jaw. Therefore, it is crucial to consider the influence of the jaw microenvironment as a whole.

The bone system is highly vascularized and sensitive to the hypoxic environment (Hart et al., 2020; Lopes et al., 2018). Within the bone microenvironment, glycolysis metabolism tends to be more active than oxidative phosphorylation (Riddle and Clemens, 2017). Additionally, the oxygen partial pressure within bones follows a distinct pattern, decreasing from the outer regions towards the inner core, with the lowest levels being less than 1%. This contrasts with the physiological range of 2%–9% observed in most tissues (Johnson et al., 2017). These unique aspects of bone metabolism create an environment that promotes tumor growth. As a result, the skeleton is not only a common site for metastasis of various primary tumors such as breast cancer, lung cancer, and prostate cancer (Sterling and Guelcher, 2011; Zhang et al., 2013), but it also provides favorable conditions for the development of malignant bone- and cartilage-forming tumors (Pierrevelcin et al., 2020).

Bone stiffness plays a significant role in the reprogramming of tumor metabolism. The growth and spread of OS, CS, and mesenchymal CS are highly sensitively to mechanical stimuli, with the RANKL/RANK, Wnt, and Hippo pathways playing crucial roles (Shoaib et al., 2022; Hsu et al., 2010; Kovar et al., 2020; Kelleher et al., 2012). RANKL is generated by various cell types, including osteocytes, osteoblasts, and immune cells. When it binds to the RANK receptor, it triggers the differentiation and activation of osteoclasts. The RANKL/RANK pathway regulates bone development, remodeling, and is implicated in bone-related disorders like osteoporosis and tumors (Rao et al., 2018; Ono et al., 2020; Rachner et al., 2011). Moreover, multiple tumor cells, including OS, can produce RANK, leading to an increase in RANKL expression (Kukita and Kukita, 2013; Tan et al., 2011) and changes in the metabolic regulator MAPK expression and mitochondrial homeostasis of tumor cells (Yuan et al., 2020; Rao et al., 2017).

Extensive research has been conducted on the role of the Wnt pathway in tumors. In addition to its role in maintaining bone homeostasis (Liu et al., 2022), the Wnt pathway plays a crucial part in stiffness-dependent immune cell polarization, tumor cell stemness, and the epithelial-mesenchymal transition during tumor development (Chen M. et al., 2020; Tao et al., 2021; Xu et al., 2021). Within the skeletal system, the Wnt pathway regulates redox signaling to maintain the necessary ecological niche for stem cell differentiation. It promotes osteogenic differentiation through processes such as glycolysis, fatty acid oxidation, and glutamine consumption while inhibiting osteoclast differentiation, ultimately leading to increased bone mass and strength (Miallot et al., 2021; Wang et al., 2015; Maeda et al., 2019; Baron and Kneissel, 2013; Shen et al., 2021). In the presence of tumors, the effects of the Wnt pathway extend beyond the remodeling of the surrounding bone. Studies have revealed crosstalk between the Wnt pathway and other pathways, including the Hippo pathway. This crosstalk contributes to tumor metabolic reprogramming by regulating the mitochondrial kinase pyruvate dehydrogenase kinase (PDK)-1 and increasing the expression of glycolysis enzymes. These changes enhance the Warburg effect, promote angiogenesis, and regulate glutamine metabolism, driving the epithelial-mesenchymal transition (Pate et al., 2014; Sebestyén et al., 2021; Lee et al., 2017; Sun et al., 2022; Adebayo Michael et al., 2019). It is important to note that tumor cells and surrounding normal cells exhibit varying sensitivities to microenvironmental stiffness. Jiang et al. conducted a study demonstrating that Wnt/β-catenin-mediated proliferation in CS cells is more responsive to matrix stiffness compared to osteoblasts, accompanied by high expression of HIF (Jiang et al., 2019).

In addition to its collaboration with the Wnt pathway, the Hippo pathway can independently regulate tumor metabolism. The Hippo pathway serves as a key regulator of tissue and organ homeostasis and size (Hansen et al., 2015; Li et al., 2021a). Molina, Coughlin, and others have shown that OS cells respond to increased matrix stiffness by upregulating the expression and nuclear translocation of YAP/TAZ (Molina et al., 2019; Coughlin et al., 2021). The activation and nuclear translocation of YAP/TAZ depend on glucose, lipid, and hormone levels (Miallot et al., 2021; Mo et al., 2015; Koo and Guan, 2018). Simultaneously, YAP/TAZ can directly interact with HIF-1α or promote GLUT3 transcription to enhance tumor glycolysis (Zhang et al., 2018; Cosset et al., 2017). Furthermore, YAP/TAZ can mediate the uptake of leucine and the catabolism of glutamic acid through the TEAD transcription factor (Hansen et al., 2015; Yang et al., 2018). The Hippo pathway also regulates tumor lipid metabolism, with YAP/TAZ promoting fat accumulation through AKT signaling (Jeong et al., 2018; Liu et al., 2019). However, there is currently no direct evidence linking the progression of malignant bone- and cartilage-forming tumors with local metabolic changes and spatial heterogeneity of stiffness. This could be a crucial area for exploration to better understand the characteristics of these types of tumors in the jaw.

### Spatial heterogeneity of cartilage stiffness and tumor metabolism

Compared to other fibrocartilage, the articular disc of TMJ exhibits lower solute diffusivities, higher oxygen consumption rates, and more significant changes in glucose concentration gradients. This difference renders the articular disc of TMJ more susceptible to pathological changes that hinder nutrition supply, such as sustained mechanical loading caused by jaw clenching and bruxism (Cisewski et al., 2015). Previous studies have mainly focused on the role of mechanical signals in TMJ’s normal physiological functioning during chewing and its association with TMJ disorders and inflammation. These studies have explored how the mechanical properties of the cartilage microenvironment affect cellular processes and pathological activities, including chondrogenic differentiation, chondrocyte proliferation and diffusion, and macrophage polarization (Bachmann et al., 2020; Chen C. et al., 2014; Donahue et al., 2022). However, limited research exists regarding the impact of mechanical properties of cartilage on tumors.

Cartilage is a hypoxic tissue that lacks a capillary network and is tightly regulated by the HIF family. In physiological conditions, HIF-1α plays a beneficial role in chondrogenic differentiation (Fernández-Torres et al., 2017; Ito et al., 2021) and contributes to the formation of cartilage ECM (Taheem et al., 2020). However, hypoxia signaling also promotes tumor growth and metastasis (Laitala and Erler, 2018). For instance, hypoxia induces the upregulation of HIF-1α and VEGF in CS cells as well (Lin et al., 2004). HIF-2α is a key regulator in the progression of OS. A study by Dietmar W. Hutmacher et al. found higher expression of HIF-2 in OS of TMJ compared to OS of limb (Wagner et al., 2019). Additionally, emerging evidence suggests that ECM stiffness affects the expression level of HIF. Researchers such as Jing Zhang have shown a positive correlation between HIF-1α and matrix stiffness in breast cancer, while it is negatively correlated with oxygen content (Zhang et al., 2020). Valerie M. Weaver et al. found that high matrix stiffness can induce HIF-1 production in two-dimensional culture conditions of glioma cells, whereas a softer matrix weakens tumor cells’ perception of hypoxia (Miroshnikova et al., 2016). Fan Yang et al.'s study highlighted that, under three-dimensional culture conditions, an increase in matrix stiffness from 40 Pa to 26.6 kPa led to reduced tumor cell proliferation but significantly upregulated HIF-1α expression along with VEGF expression (Wang et al., 2021). Although these findings provide evidence for the association between matrix stiffness, hypoxia, and tumors, the sensitivity of tumor cells and tumor-associated cells in TMJ cartilage to stiffness may vary, and further research is needed to explore these relationships.

### Temporal heterogeneity of bone stiffness and tumor metabolism

The incidence rate, therapeutic effect and prognosis of malignant tumors are age dependent. OS, for instance, exhibits a bimodal age distribution, with peak incidences in adolescence and older adults. Different age groups also display varying sensitivities to chemotherapy, with poorer prognoses observed in older adults (Ottaviani and Jaffe, 2009; Wu and Huang, 2015). CS, on the other hand, is more commonly diagnosed in individuals aged 50–70, and younger patients tend to have better prognoses (Xie et al., 2022). However, a specific subtype known as mesenchymal CS has a higher tendency to occur in young people (Flaman et al., 2020; Choo et al., 2019). It should be noted, though, that further research is needed to establish a direct connection of these age-related differences between tumors and the material characteristics of tissues.

Age-related changes in tissue stiffness are closely linked to the remodeling and accumulation of ECM components, including but not limited to collagen and laminin (Angelidis et al., 2019; Lofaro et al., 2021; Ryu et al., 2021). As bones mature and age, there are notable modifications in the molecular composition, quantity, cross-linking structure, and arrangement of type I collagen, which ultimately contribute to alterations in the material characteristics of tissues (Kaku et al., 2007; Van Gulick et al., 2022; Kwansa et al., 2016; Guilbert et al., 2014). The age-dependent modifications in collagen have a direct impact on the occurrence and progression of tumors. For instance, collagen glycosylation can regulate the interact site between tumor cells and type I collagen (Guilbert et al., 2014), inhibiting the activation of the DDR1 site and thereby affecting tumor cell proliferation (Saby et al., 2018). Furthermore, collagen glycosylation presents an obstacle to the degradation of the ECM mediated by MMPs (Bartling et al., 2009; Panwar et al., 2018). The MMP-mediated degradation of ECM not only influences its stiffness but also indirectly regulates the migration of tumor cells and tumor-associated cells, accompanied by the influence on the generation of oxidative stress-related reactive oxygen species (ROS) and the accumulation of glycation end products (AGE) (Guilbert et al., 2014; Kaur et al., 2019; Worrede et al., 2021; Panwar et al., 2015; Komsa-Penkova et al., 2022).

An increasing body of evidence has demonstrated the role of AGE as a promoter in the occurrence and progression of tumors. AGE not only contributes to local inflammation and facilitates tumor invasion and metastasis (Dariya and Nagaraju, 2020; Schröter and Höhn, 2018), but it may also induce the transformation of healthy cells into tumor cells through protein aggregation (Haque et al., 2019). Moreover, metabolites associated with tumors can interact with the ECM of surrounding tissues, leading to changes in tissue stiffness. For instance, studies have reported that poly (ADP-ribosyl) ated RPA can interfere with bone mineralization (Hegedus et al., 2015). Age-related alterations in collagen structure and impaired clearance of glycoprotein further affect the metabolism of AGE (Haque et al., 2019). Meanwhile, AGE attacks collagen and elastin, resulting in ECM remodeling (Fournet et al., 2018). Considering the above findings, the age distribution pattern of malignant bone- and cartilage-forming tumors may be associated with the temporal heterogeneity of tissue stiffness, warranting further research in the future.

### Temporal heterogeneity of cartilage stiffness and tumor metabolism

The metabolic disorder is one of signs of aging and degenerative diseases in cartilage, characterized by a decrease in oxygen levels and fluctuations in glucose concentration (Cisewski et al., 2015). The permanent degradation of TMJ can result from wear, primarily caused by excessive shear loading. As joint wear and changes in cartilage material properties occur, there is an increase in HIF-1α and VEGF (Kuroda et al., 2009). Research has explored the destruction of joint lubrication, facilitated by enzyme catalysis, limits the friction characteristics of condylar cartilage. This contributes to the progression of degenerative TMJ lesions, evidenced by elevated levels of cyclooxygenase (COX)-2 and MMPs, as well as the loss of type II collagen under cyclic loading (Hill et al., 2014; Asakawa-Tanne et al., 2015).

COX-2 is a mechanically sensitive protein. Excessive COX-2 can mediate the production of its metabolite prostaglandin E_2_, which further exacerbates hypoxia in chondrocytes. Additionally, alterations in shear force have been found to regulate the expression of COX-2 in CS cells (Branco da Cunha et al., 2014; Su et al., 2017). Significantly, COX-2 and its metabolites can work in synergy with other mechanically sensitive proteins like YAP to reduce apoptosis in tumors and tumor-associated cells. They also promote proliferation, angiogenesis, inflammation, metastasis, and enhance tumor resistance to chemotherapy (Hashemi G et al., 2019).

In addition, recent studies have shown that during joint maturation and aging, there is an increase in mitofusin (MFN)-2, a mechanical sensitive protein involved in mitochondrial fusion in chondrocytes. This increase in MFN-2 is accompanied by a shift in metabolic mode towards mitochondrial respiration, leading to increased oxygen consumption (Zarei et al., 2021; Xu et al., 2020). MFN-2 plays an important role in facilitating mitochondrial respiratory stress adaptation and the production of ROS and reactive nitrogen species such as nitric oxide, which are necessary for maintaining immune cell function (Lloberas et al., 2020). However, knockout of MFN-2 may also facilitate glucose uptake by chondrocytes and reverse age-related metabolic changes (Zarei et al., 2021; Xu et al., 2020; Bartolak-Suki et al., 2015). Furthermore, previous research has demonstrated that changes in chondrocyte metabolism not only affect the proportion of ECM components but also directly participate in AGE-mediated abnormal collagen synthesis through MFN-2 (Ozanturk et al., 2016). These findings suggest that altered MFN-2 activity may be one of the contributing factors influencing tumor development, treatment, and prognosis of TMJ disorders.

## Stiffness biomimetic scaffolds and their implication in the research of malignant bone- and cartilage-forming tumors

The selection of stiffness biomimetic scaffold manufacturing technology depends on its ability to achieve stiffness compatibility with the target tissue. While two-dimensional culture systems remain widely used for studying tumor molecular mechanisms due to their mature construction techniques and ease of use, three-dimensional culture systems may offer better simulation of the *in vivo* tumor growth environment.

### Strategies for the construction of stiffness biomimetic scaffolds

**FIGURE 2:**
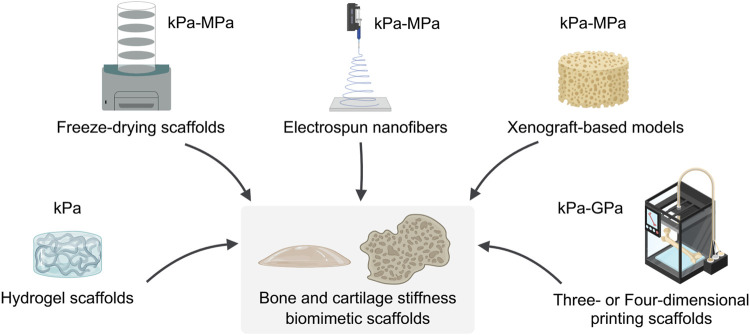
Fabrication and mechanical properties of bone and cartilage biomimetic scaffolds. The technique of constructing bone and cartilage biomimetic scaffolds and the range of stiffness of the resulting biomimetic scaffolds. This figure created with MedPeer.cn.

As illustrated in Figure 2, the mainstream strategies in three-dimensional culture systems for investigating the correlation between stiffness and tumors include hydrogel scaffolds, Freeze-drying scaffolds, electrospun nanofibers, Xenograft-based models, and three- or four-dimensional printing scaffolds.

#### Hydrogel scaffolds

Hydrogels are widely used for constructing *in vitro* tumor models owing to their highly adjustable stiffness, which can be modulated through crosslinking methods, material composition, and the incorporation of additives such as nanomaterials (Caliari and Burdick, 2016; Cao et al., 2021; Li and Kumacheva, 2018). For instance, composite alginate-Matrigel hydrogels enable independent control over matrix stiffness and composition. By adjusting the degree of ionic cross-linking of alginate with Ca^2+^ ions while maintaining constant polymer concentration, ligand density, and pore size, the stiffness of these hydrogels can be precisely tuned (Li and Kumacheva, 2018). Furthermore, the incorporation of nanobioactive glass has been shown to enhance the compressive modulus of collagen gel by more than tenfold (Marel et al., 2011), while nanoclay-peptide co-assembly facilitates the fabrication of hydrogels possessing high stiffness and self-healing properties (Okesola et al., 2021). Aramid nanofiber-based hydrogels also exhibit high modulus and strength (Sun et al., 2023), underscoring the potential of nanomaterials in developing stiffness-tunable biomimetic platforms. The development of stiffness gradient hydrogels (Hakeem et al., 2023) and heterogeneous hydrogel arrays (Yue et al., 2018) further facilitate systematic analysis of stiffness effects on tumor behavior within a unified experimental system, thereby reducing inter-experimental variability. It should be noted, however, that although hydrogels can effectively mimic softer tissues such as breast or brain, they are typically limited to stiffness values below 100 kPa (Jabbari et al., 2015; Chaddad et al., 2017; De Luca et al., 2018), a range that supports cell viability but remains considerably lower than that characteristic of bone tissue. Therefore, hydrogel-based systems may be better suited for constructing cartilage stiffness biomimetic scaffolds, as the stiffness range of cartilage falls within what can be achieved with hydrogels. However, if hydrogel-based materials are to be used for replicating bone-like stiffness, they need to be combined with stiffer scaffold systems to achieve the required mechanical properties.

#### Freeze-drying scaffolds

Freeze-drying is a widely used method for fabricating composite scaffolds, largely because it can produce three-dimensional structures with high porosity and interconnected pore networks. These features closely resemble the natural structure of bone and cartilage tissues (Marturano-Kruik et al., 2018). The mechanical properties of such scaffolds can be regulated by adjusting their composition and applying crosslinking strategies. For example, scaffolds incorporating bioactive elements such as deferoxamine-loaded microchannels have achieved a Young’s modulus of 0.32 MPa while maintaining good cytocompatibility (Xue et al., 2025). Similarly, the introduction of cerium ions into piezoelectric scaffolds with controlled Mg^2+^ release has led to compressive strengths of up to 2.89 MPa (Kang et al., 2025). Stiffness can also be improved by modifying polymer ratios and using chemical crosslinking. This is demonstrated in polycaprolactone (PCL)-gelatin systems, where compressive strength can be adjusted from 10 to 60 MPa (Badami et al., 2025), and in chemically crosslinked chitosan-hyaluronic acid scaffolds, which show enhanced stiffness and slower degradation (Hamidi et al., 2025). However, scaffolds produced via freeze-drying generally exhibit stiffness values ranging from kPa to MPa, which remains below that of native bone tissue. Although freeze-dried scaffolds are suitable for cartilage-like applications, they are still insufficient for replicating the mechanical environment of bone. Future research could focus on combining freeze-drying with other fabrication techniques or incorporating reinforcement materials to extend its use toward developing biomimetic scaffolds with bone-like stiffness.

#### Electrospun nanofibers

Electrospinning technology is frequently combined with other manufacturing strategies to create scaffolds that simulate the porous structure of native bone and cartilage. Recently, a research team has assembled short nanofibers containing porous silica nanoparticles with a three-dimensional printed HA/PCL scaffold to create a scaffold with strong compressive strength and an adjustable porous structure (Zhou et al., 2023). Beyond simulating tissue porosity, electrospun technology can replicate the micro/nanofibrous architecture of the natural ECM (Li et al., 2021b). By controlling fiber composition, morphology, and alignment, the stiffness of the scaffolds can be systematically enhanced to the order of MPa. Furthermore, there has been a growing use of metal and carbon-based materials in addition to natural and synthetic materials for producing nanofibers, further enhancing the stiffness of scaffolds (Chen et al., 2018; A et al., 2022; Erickson et al., 2022). However, one limitation is that it is challenging to achieve a stiffness level of GPa, which is closer to that of bone, with electrospun nanofiber scaffolds.

#### Xenograft-based models

The study and application of xenograft-based models have advanced in recent years. One notable development involves the use of demineralized cortical bone from bovine femurs as a scaffold, with a thickness of 20 μm and a reported tensile modulus of 6.5 ± 0.4 kPa. This scaffold has demonstrated excellent biocompatibility for cell culture (Park et al., 2021). In another study, a scaffold derived from demineralized porcine femoral heads was used to investigate chemical drug resistance in OS cells (Ren et al., 2024). The compressive modulus of this decellularized bone matrix scaffold was measured at 15.68 ± 1.39 MPa in the dry state and 1.44 ± 0.06 MPa in the wet state. These examples suggest that future studies could employ bone-derived scaffolds from different sites to examine how spatial heterogeneity of bone stiffness influences tumor behavior. It should be noted that while such models provide a promising approach for exploring tissue-level mechanical effects on tumors, achieving precise control over stiffness in these systems remains a challenge and requires further investigation.

#### Three-dimensional/Four-dimensional printing scaffolds

Three-dimensional printing technology offers the advantage of faithfully replicating the morphological structure of tissues and organs, while also allowing for the simulation of gradient changes in ECM composition and stiffness (Bittner et al., 2019). Building upon this foundation, recent advancements in four-dimensional printing have enabled dynamic regulation of structure and mechanical properties in response to environmental changes (Wan et al., 2020). Although natural bioprinting inks demonstrate favorable biocompatibility, their mechanical properties are often insufficient (Jung et al., 2018). In comparison, synthetic polymer composites and ceramic-based scaffolds demonstrate substantially enhanced mechanical performance while maintaining biocompatibility, making them promising materials for applications in tumor research models (Bittner et al., 2019; Xu et al., 2024). Metallic materials are capable of replicating high-stiffness microenvironments (Niinomi et al., 2016; Prasad et al., 2017; Dewey and Harley, 2021); nevertheless, their smooth surfaces do not promote cell adhesion and growth as effectively as natural materials. Other research teams and our previous studies have highlighted the significance of rough micro/nano topography on material surfaces in influencing cell behavior (Kumar et al., 2020; Li et al., 2019). Notably, three-dimensional printing can generate disordered micro/nano structures on the metal surface, as observed in our previous series of studies (Wang et al., 2018; Wang et al., 2019). This structural feature may facilitate cell adhesion and offer potential advantages in constructing a biomimetic scaffold with bone-like stiffness in tumor research.

### Degradability of materials

In addition to considering the stiffness, it is important to also address the degradability of materials. The degradability of materials is a double-edged sword in the study of the correlation between tissue stiffness and tumor metabolism. On one hand, material degradation accompanied by changes in stiffness can be used to explore the temporal heterogeneity of bone and cartilage, as well as their interaction with tumor metabolism. On the other hand, in studies that do not consider temporal heterogeneity, inappropriate material degradation leading to a decrease in model stiffness can result in unreliable conclusion. As showed in Table 7: (1) Degradable materials: Natural materials, such as silk, acellular ECM, and ECM components like collagen, polysaccharides, and hyaluronic acid, are widely used. In addition to natural materials, polymer materials like PCL, poly (lactic acid) (PLA), polyethylene glycol (PEG), ceramic materials like β-tricalcium phosphate (β-TCP), 45S5 bioactive glass, are also employed (Caliari and Burdick, 2016; Cao et al., 2021; Garrido et al., 2022). Some metals also possess degradability, called biodegradable metals like magnesium, zinc, iron and their alloys (Globig et al., 2020; Xia et al., 2021). It is noteworthy that although some materials may have limitations as substitutes for bone or cartilage, such as the potential for metabolic complications caused by solid iron, this does not necessarily restrict their use in tumor *in vitro* research. (2) Non- or hard-degradable materials: Ceramic materials like HA, polymer materials such as polyether (PE), polyetheretherketone (PEEK), and polyetherketoneketone (PEKK), and metals such as titanium, titanium alloys, and cobalt-chromium-molybdenum alloys, fall into this category (Zhang et al., 2019; Geevarghese et al., 2022; Hu et al., 2021; Migita et al., 2022). Although they are difficult to degrade or non-degradable, these materials effectively enhance the mechanical strength of scaffolds. Additionally, combining above materials with different degradation performance is a common strategy in tissue engineering. For instance, biphasic calcium phosphate (BCP), a blend of β-TCP and HA, has been the gold standard for bone substitutes in bone reconstruction surgery (Bouler et al., 2017). The mix of PEKK and 45S5 bioactive glass demonstrates better mechanical properties, bioactivity, and bone cell response (Garrido et al., 2022). These blended materials, with high stiffness and adjustable biodegradability, still hold vast potential for application in other tissue engineering works, such as tumor research.

**TABLE 7:** Material degradability.

Material type	Material name	Degradability	References
Natural materials	silk, acellular ECM, collagen, polysaccharides, hyaluronic acid	Yes	Caliari and Burdick (2016), Cao et al. (2021)
polymer materials	PLA, PEG, PCL	Yes	Caliari and Burdick (2016), Cao et al. (2021), Geevarghese et al. (2022)
	PE, PEEK, PEKK	No/hard	Garrido et al. (2022), Zhang et al. (2019), Hu et al. (2021), Migita et al. (2022), Geevarghese et al. (2022)
Ceramic materials	β-TCP, 45S5 bioactive glass	Yes	Garrido et al. (2022)
	HA, BCP	No/hard	Zhang et al. (2019), Bouler et al. (2017)
Metals	magnesium, zinc, iron and their alloys	Yes	Globig et al. (2020), Xia et al. (2021)
	titanium, titanium alloys, cobalt-chromium-molybdenum alloys, Tantalum	No/hard	Zhang et al. (2019), Hu et al. (2021), Migita et al. (2022), Geevarghese et al. (2022)

### Application of bone stiffness biomimetic scaffolds in the tumor research

In recent years, there has been an increasing focus on studying the influence of tissue stiffness in tumor. The suitable stiffness for tumor growth is closely related to the tissue source of tumor cells. For example, research indicated that the suitable stiffness for the growth of bone derived malignant tumor cells is greater than that of breast derived malignant tumor cells (Jabbari et al., 2015). However, there are still limitations in terms of constructing biomimetic microenvironments and setting the appropriate range of stiffness.

**FIGURE 3:**
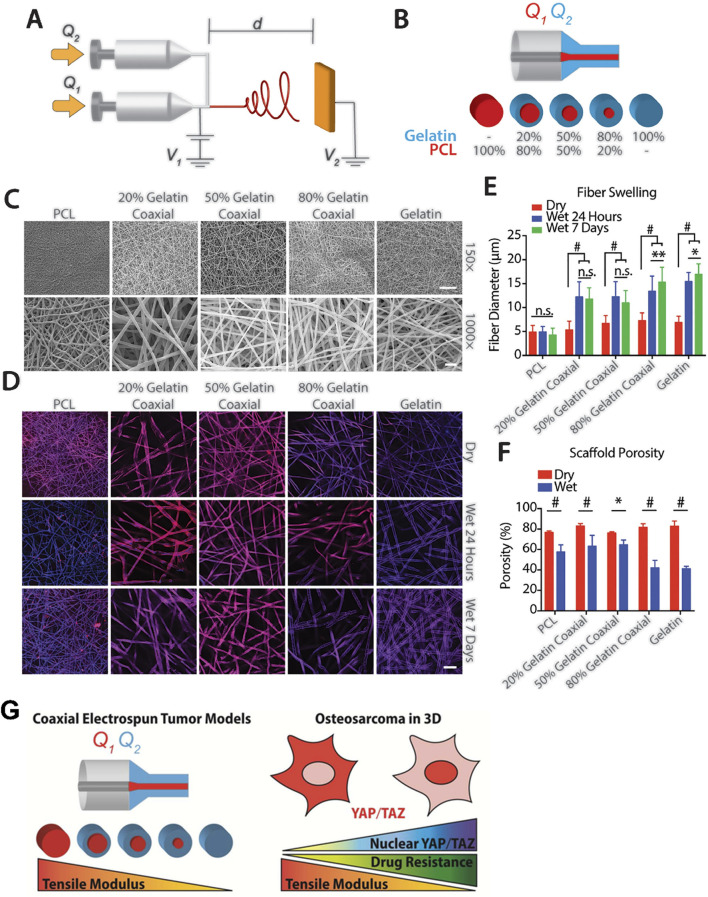
Construction of bone stiffness biomimetic scaffolds using electrospinning technology for the research of osteosarcoma. **(A)** Schematic diagram of a coaxial electrospinning device. **(B)** Schematic representation of the composition of single fibers in electrospun meshes with different PCL and gelatin ratios. **(C)** Representative electron microscopy images mesh composed of five fiber types with different core-shell PCL-gelatin compositions. **(D)** Representative confocal microscopy images of a single plane illustrating fiber swelling in an aqueous solution. Top: Dry fibers. Middle: Fibers after 24 h in aqueous solution. Bottom: Fibers after 7 days in aqueous solution, scale bar = 50 μm. **(E)** Swelling of individual fibers assessed by measuring fiber diameter over time (n = 30 fibers per group at each time point). **(F)** Porosity of meshes with variable fiber composition in dry (red) and wet (blue) conditions after 24 h of submersion in aqueous solution. n = 3. n.s. = no significance, *p < 0.05, **p < 0.01, #p ≤ 0.001. **(G)** Schematic diagram indicating an increase in the nuclear import of YAP/TAZ and drug resistance of osteosarcoma cells as the elastic modulus of the bone stiffness biomimetic scaffold decreases (Molina et al., 2019).

One study conducted by Antonios G Mikos et al. involved the construction of coaxial electrospun models with PCL and gelatin (Figure 3). They discovered that increasing the tensile modulus of the scaffold hindered the nuclear translocation of YAP/TAZ and downregulated the mTOR expression of OS cells in a three-dimensional culture environment. Not only simulated the porous structure of bone, this study elevated the tensile modulus of the scaffold to 100 MPa (Molina et al., 2019). Another research group led by Scott A. Guelcher used polyacrylamide hydrogels to mimic breast stiffness and poly (ester urethane) films to mimic stiffness ranging from the basement membrane to bone. In their two-dimensional culture system, the elastic modulus ranged from 0.45 kPa to 67 GPa. The study found that a modulus greater than 1 GPa was sufficient to upregulate PTHrP expression in osteolytic, metastatic MDA-MB-231 tumor cells, but this effect was not observed in MCF-7 cells, which do not cause osteolytic lesions. Additionally, when the elastic modulus of the ECM reached the order of GPa, further changes in PTHrP levels were no longer significant (Ruppender et al., 2010; Sterling and Guelcher, 2011). Based on the aforementioned findings, the research group subsequently narrowed down the range of ECM elastic modulus to between 0.07 GPa and 3.8 GPa for their subsequent studies (Page et al., 2015). This series of studies emphasized the importance of tissue stiffness in tumor development. However, the growth and metabolic patterns of cells in two-dimensional and three-dimensional culture environments are not entirely consistent. For instance, Tingting Tang et al.'s study demonstrated that OS cells exhibit stronger catabolism in a three-dimensional culture environment compared to a two-dimensional culture, making them more sensitive to chemotherapy drugs that target autophagy pathways (Lin et al., 2022). It is worth mentioning that the stiffness range setting in this study was based on the stiffness of the tumor itself rather than the stiffness of the tissue in which the tumor grows. Similarly, Oran D Kennedy et al. investigated the regulatory effect of malignant bone tumor stiffness on the SOX2-YAP signal axis (Coughlin et al., 2021).

**FIGURE 4:**
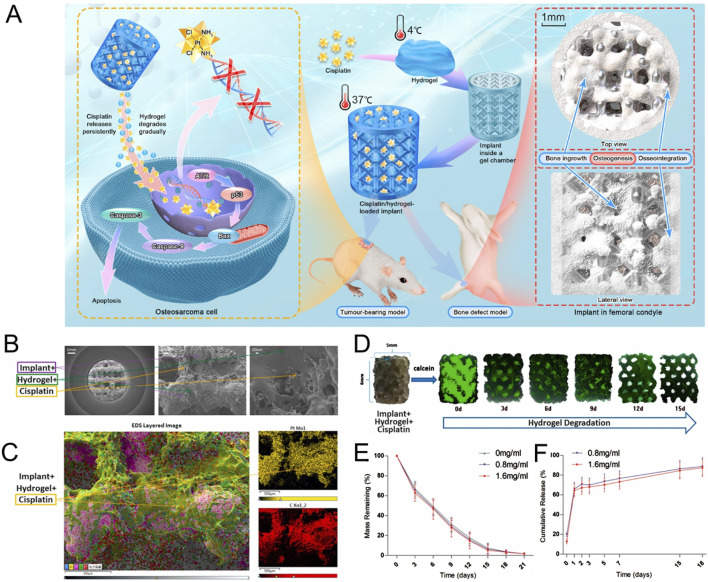
Construction of anti-osteosarcoma bone substitutes using three-dimensional printing technology. **(A)** Schematic illustration of the method for preparing cisplatin/hydrogel-loaded three-dimensional-printed Ti6Al4V implants, which exhibit anti-tumor and bone-repairing effects. **(B)** Representative electron microscopy images of cisplatin/hydrogel-loaded Ti6Al4V implants. **(C)** Representative energy dispersive spectroscopy layer images showing cisplatin integration into the hydrogel. **(D)** *In vitro* degradation of the hydrogel (green) incorporated into the implants (0–15 days). **(E)** Mass remaining of hydrogel (0–21 days). **(F)** *In vitro* release profile of cisplatin from the cisplatin/hydrogel-loaded Ti6Al4V implants (0–18 days). n = 3 (Jing et al., 2021).

Although the stiffness range of *in vitro* models in the field of bone tumor mechanism research is wide, there appears to be a consensus regarding the research and application of scaffolds for repairing bone defects, particularly large area defects. The aim is to closely match the structure and mechanical properties of the scaffolds with those of bone tissue. This provides valuable insights for the development of *in vitro* models of bone tumors. The stiffness of the scaffold made from metals such as titanium and magnesium, and their alloys, can easily reach the GPa level (Niinomi et al., 2016; Prasad et al., 2017; Dewey and Harley, 2021). Zhongjun Liu’s team recently constructed a three-dimensional printing titanium alloy scaffold combined with cisplatin-loaded hydrogel (Figure 4). This scaffold can not only repair bone defects but also have the potential to resist OS (Jing et al., 2021). Bérengère J.C. Luthringer-Feyerabend et al. presented a study investigating the anti-tumor potential of magnesium-based biomaterials. Considering that the tensile strength of magnesium and magnesium alloys is similar to that of normal bone cortex (Globig et al., 2020), they show promise for using in the exploration of the impact of bone stiffness on tumors.

**FIGURE 5:**
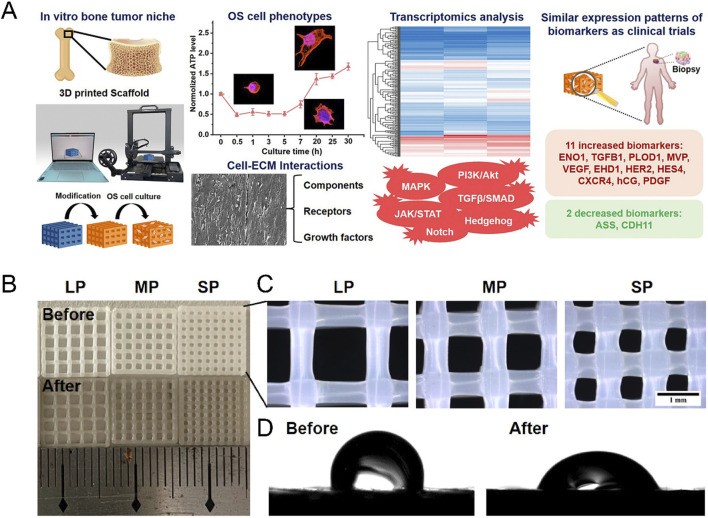
Construction of bone stiffness biomimetic scaffolds using three-dimensional printing technology for the research of osteosarcoma. **(A)** Schematic illustration of the development of bone stiffness biomimetic scaffolds from PLLA, fabricated by three-dimensional printing technology, for *in vitro* three-dimensional culture of osteosarcoma cells. Cells cultured in this scaffold exhibit characteristics closer to those of osteosarcoma patient biopsy samples. **(B)** Representative photographs of PLLA scaffolds. **(C)** Representative stereomicroscope images of PLLA scaffolds. BP (big pore), MP (medium pore), SP (small pore). **(D)** Water contact angles of PLLA scaffolds with and without dopamine coating (Wang ML. et al., 2022).

In addition to metals, polymer materials also show promise in constructing *in vitro* models of tumors. Bittner SM et al. printed a scaffold similar to the trabecular bone compression modulus by combining PCL with nano HA (Bittner et al., 2019). A recent study conducted by Xiqiu Liu et al. employed poly (l-lactide) (PLLA) as the raw material to fabricate an OS model using three-dimensional printing technology (Figure 5). The compressive strength of the constructed model surpassed 100 MPa, closely resembling the cortical bone (Wang ML. et al., 2022). This exemplifies the capability of polymer-based materials and the three-dimensional printing technology in generating *in vitro* models that mimic the mechanical properties of bone.

### Application of cartilage stiffness biomimetic scaffolds in the tumor research

Upon searching the database, we have found that existing research on tumors in the cartilage region primarily relies on the two-dimensional culture model. Three-dimensional culture model usually using spheres and organoids made from materials like matrix glue and alginate (Voissiere et al., 2017; Veys et al., 2021; Palubeckaitė et al., 2020), which are unable to effectively simulate the mechanical properties of the cartilage surrounding the tumor.

**FIGURE 6:**
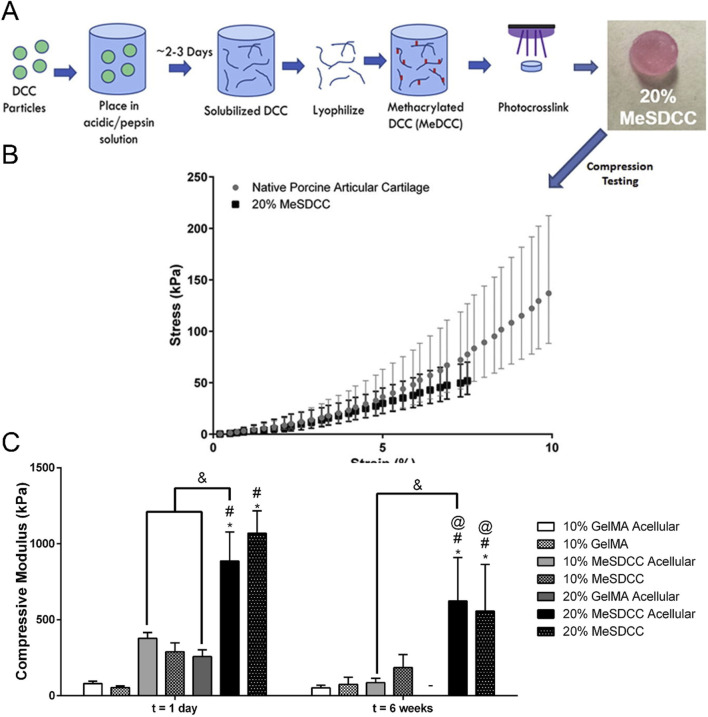
Construction of cartilage stiffness biomimetic decellularized cartilage-based hydrogel scaffolds for three-dimensional cell culture. **(A)** Schematic illustration of the methacrylated solubilized decellularized cartilage (MeSDCC) hydrogels synthesis process. **(B)** Stress-strain curves of native porcine cartilage and hydrogels. Data are reported as mean ±95% confidence interval. The results show that the 20% MeSDCC hydrogels fell within the 95% confidence interval of native porcine cartilage until it begins to fracture at an average strain of 7.5%. **(C)** Compressive modulus of hydrogels with or without rat bone marrow stems cells encapsulated shows that 20% MeSDCC hydrogels with cells encapsulated had similar modulus to porcine cartilage (Beck et al., 2016).

**FIGURE 7:**
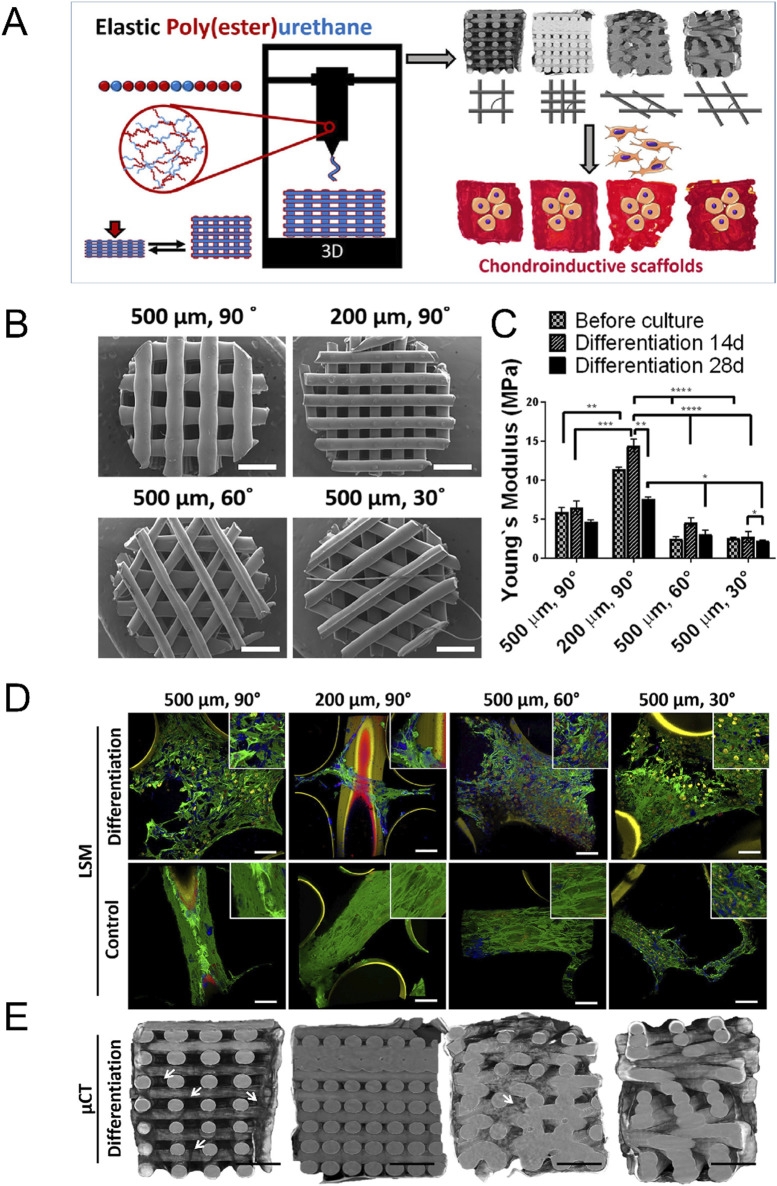
Construction of cartilage stiffness-mimicking poly(ester)urethane (PEU) scaffolds using three-dimensional printing technology for three-dimensional cell culture. **(A)** Schematic representation of the PEU scaffold manufacturing process. These scaffolds are designed for the three-dimensional culture of osteosarcoma cells (ATDC5) *in vitro*. **(B)** Representative scanning electron microscope images of PEU scaffolds with varying pore sizes and deposition patterns. **(C)** Non-equilibrium compressive Young’s modulus of scaffolds in PBS, before and after 14 and 28 days of culture in differentiation media. Scaffolds with a 200 µm pore size and a 90° deposition pattern exhibit a modulus of approximately 10 MPa. Data are presented as mean ± SD, n = 3. **(D)** Representative light scanning microscopy images of scaffolds after culture in differentiation and basal media for 28 days. Green indicates F-Actin, Yellow indicates nuclei, Blue indicates collagen II, and Red indicates collagen I. **(E)** Representative micro-X-ray computed tomography reconstructions of scaffolds after culture in differentiation media for 28 days. The images demonstrate ATDC5 cell infiltration and ECM deposition within the scaffold pores (Camarero-Espinosa et al., 2020).

Some biomimetic models of cartilage stiffness in existing research have successfully elevated the scaffold moduli to a range close to cartilage stiffness, which may have application prospects in the research of malignant bone- and cartilage-forming tumors. The electrospun gelatin/monetite nanofibrous scaffold prepared by Yogendra Pratap Singh et al. was found that when the monetite content increased, its tensile strength and modulus exceeded 10 MPa, and promoted MG-63 cell adhesion, proliferation, higher bio-mineralization and Alkaline phosphatase activity (Singh et al., 2023). Hydrogel is the most commonly used material to simulate cartilage. For instance, Melissa A Grunlan and her team developed a triple-network polymer hydrogel. By incorporating electrostatic repulsive and attractive interactions, along with hydrophobic interactions, they successfully enhanced the compressive and tensile moduli of scaffolds to a range of 1.5 MPa–3.5 MPa, closely resembling moduli of natural cartilage (Demott et al., 2022). Michael S Detamore, et al. successfully developed methacrylated solubilized decellularized cartilage (MeSDCC) hydrogels with an elastic compression modulus of nearly 1 MPa when cells were encapsulated (Figure 6). This three-dimensional culture model effectively promotes the upregulation of chondrogenic differentiation in bone marrow stem cells (Beck et al., 2016). Besides, as showed in Figure 7, Lorenzo Moroni and teams using fused deposition to build three-dimensional printing fabricated a poly(ester)urethane (PEU) scaffold with different pore sizes, which showed matched Young’s modulus (10 MPa) to cartilage, and showed chondrocyte cells ATDC5 infiltration and ECM deposition in pores (Camarero-Espinosa et al., 2020).

## Outlook

We emphasize that in the construction of *in vitro* models of tumors, it is necessary to fully consider that tumor growth and invasion involve multiple tissues with different material properties simultaneously or sequentially, along with significant variations in metabolic types and levels. In addition to the mentioned stiffness heterogeneity of bone and cartilage, it is also essential to consider the stiffness heterogeneity of maxillofacial soft tissues and the impact of tumor body stiffness on tumor metabolism when constructing *in vitro* models.

Based on these considerations, we believe that utilizing a combination of manufacturing strategies to develop a tissue stiffness biomimetic model for tumor research is a promising direction for future studies. By capitalizing on the distinct features of various manufacturing techniques and the adjustable properties of raw materials, we can simulate the spatial heterogeneity of tissue stiffness. Additionally, incorporating material degradation processes allows us to replicate the temporal heterogeneity of tissue stiffness. This comprehensive approach will contribute to a deeper understanding of the relationship between metabolic reprogramming and tissue stiffness during the onset and progression of tumors, thereby contributing to more precise diagnosis and effective treatment of malignant bone- and cartilage-forming tumors in the jaw and TMJ.
